# Concordance of genetic variation that increases risk for Tourette Syndrome and that influences its underlying neurocircuitry

**DOI:** 10.1038/s41398-019-0452-3

**Published:** 2019-03-22

**Authors:** Mary Mufford, Josh Cheung, Neda Jahanshad, Celia van der Merwe, Linda Ding, Nynke Groenewold, Nastassja Koen, Emile R. Chimusa, Shareefa Dalvie, Raj Ramesar, James A. Knowles, Christine Lochner, Derrek P. Hibar, Peristera Paschou, Odile A. van den Heuvel, Sarah E. Medland, Jeremiah M. Scharf, Carol A. Mathews, Paul M. Thompson, Dan J. Stein, Psychiatric Genomics Consortium - Tourette Syndrome working group

**Affiliations:** 1 Human Genetics Research Unit, Division of Human Genetics, Department of Pathology, Institute of Infectious Disease and Molecular Medicine, Faculty of Health Sciences, University of Cape Town, Cape Town, South Africa; 2 Imaging Genetics Center, Mark and Mary Stevens Neuroimaging & Informatics Institute, Keck School of Medicine of the University of Southern California, Los Angeles, CA USA; 3 Department of Psychiatry and MRC Unit on Risk & Resilience, University of Cape Town, Cape Town, South Africa; 4 Groote Schuur Hospital and Neuroscience Institute, Cape Town, South Africa; 5 Department of Psychiatry and the Behavioural Sciences, Zilkha Neurogenetic Institute, Keck School of Medicine, University of Southern California, Los Angeles, CA USA; 6 Department of Psychiatry, University of Stellenbosch, Stellenbosch, South Africa; 7 Department of Biological Sciences, Purdue University, West Lafayette, IN USA; 8 Department of Psychiatry, Department of Anatomy & Neurosciences, VU University Medical Center, Amsterdam Neuroscience, Amsterdam, Netherlands; 9 QIMR Berghofer Medical Research Institute, Brisbane, Australia; 10 Psychiatric and Neurodevelopmental Genetics Unit, Center for Genomic Medicine, Harvard Medical School, Boston, MA USA; 11 Department of Psychiatry, Massachusetts General Hospital, Boston, MA USA; 12 Department of Neurology, Massachusetts General Hospital, Boston, MA USA; 13 Department of Psychiatry, Genetics Institute, University of Florida, Gainesville, FL USA

**Keywords:** Comparative genomics, Psychiatric disorders, Clinical genetics, Neuroscience

## Abstract

There have been considerable recent advances in understanding the genetic architecture of Tourette Syndrome (TS) as well as its underlying neurocircuitry. However, the mechanisms by which genetic variation that increases risk for TS—and its main symptom dimensions—influence relevant brain regions are poorly understood. Here we undertook a genome-wide investigation of the overlap between TS genetic risk and genetic influences on the volume of specific subcortical brain structures that have been implicated in TS. We obtained summary statistics for the most recent TS genome-wide association study (GWAS) from the TS Psychiatric Genomics Consortium Working Group (4644 cases and 8695 controls) and GWAS of subcortical volumes from the ENIGMA consortium (30,717 individuals). We also undertook analyses using GWAS summary statistics of key symptom factors in TS, namely social disinhibition and symmetry behaviour. SNP effect concordance analysis (SECA) was used to examine genetic pleiotropy—the same SNP affecting two traits—and concordance—the agreement in single nucelotide polymorphism (SNP) effect directions across these two traits. In addition, a conditional false discovery rate (FDR) analysis was performed, conditioning the TS risk variants on each of the seven subcortical and the intracranial brain volume GWAS. Linkage disequilibrium score regression (LDSR) was used as validation of the SECA method. SECA revealed significant pleiotropy between TS and putamen (*p* = 2 × 10^−4^) and caudate (*p* = 4 × 10^−4^) volumes, independent of direction of effect, and significant concordance between TS and lower thalamic volume (*p* = 1 × 10^−3^). LDSR lent additional support for the association between TS and thalamus volume (*p* *=* 5.85 × 10^−2^). Furthermore, SECA revealed significant evidence of concordance between the social disinhibition symptom dimension and lower thalamus volume (*p* = 1 × 10^−3^), as well as concordance between symmetry behaviour and greater putamen volume (*p* = 7 × 10^−4^). Conditional FDR analysis further revealed novel variants significantly associated with TS (*p* < 8 × 10^−7^) when conditioning on intracranial (rs2708146, *q* = 0.046; and rs72853320, *q* = 0.035) and hippocampal (rs1922786, *q* = 0.001) volumes, respectively. These data indicate concordance for genetic variation involved in disorder risk and subcortical brain volumes in TS. Further work with larger samples is needed to fully delineate the genetic architecture of these disorders and their underlying neurocircuitry.

## Introduction

Tourette’s Syndrome (TS) has a global prevalence of ~0.85–1%^1^ and is characterised by repetitive motor and phonic tics, with onset typically before the age of 18 years^2^. TS has one of the highest heritability estimates for neuropsychiatric disorders (70–80%)^3^, with 50–60% of this heritability directly attributable to single nucleotide polymorphisms (SNPs)^4^. In recent years, there have been significant advances in understanding the genetic architecture of TS and in delineating other aspects of its underlying neurobiology, including its specific neuroanatomy^1^.

The Psychiatric Genomics Consortium Tourette Syndrome working group (PGC-TS) undertook the first genome-wide association study (GWAS) of TS, comprising 1285 cases and 4964 controls^5^. While no SNP reached genome-wide significance, the top-ranking variants were enriched for genes that affect gene expression and methylation levels in the fronto-striatal circuitry, consistent with contemporary models of TS^6^. The lack of genome-wide significance at this sample size likely reflects the polygenic and heterogeneous nature of TS^7–9^, which is further complicated by comorbidity with other psychiatric disorders, such as obsessive-compulsive disorder (OCD), autism spectrum disorders (ASD) and attention-deficit/hyperactivity disorder (ADHD)^10^. Another study used the most highly associated variants from the PGC-TS GWAS to predict TS status (*p* = 0.042) in an independent cohort (609 cases and 610 controls) and they accounted for 0.52% of the variance observed between cases and controls^11^.

Several studies have attempted to clarify the complex nature of TS by identifying more homogenous endophenotypes and symptom dimensions^12–15^. While these studies identified several classes of Tourette-related endophenotypes using multivariate methods, all were based on relatively small samples (*n* < 1000)^13–15^. A recent and considerably larger analysis of individuals with TS and their family members that assessed not only TS, but also OCD and ADHD (*n*_total_ = 3494), identified two cross-disorder symptom dimensions, namely social disinhibition and symmetry behaviour^16^. Social disinhibition includes uttering syllables/words, echolalia/palilalia, coprolalia/copropraxia, and obsessive urges to offend/mutilate/be destructive. Symmetry behaviour includes symmetry, evening up, checking obsessions, ordering, arranging, counting, writing-rewriting compulsions and repetitive writing tics. Social disinhibition (*h*^2^ = 0.35 ± 0.03) was associated with OCD polygenic risk scores (PRS; *p* = 0.02) and less strongly with TS and ADHD PRS, which did not meet statistical significance (*p* = 0.11 and *p* = 0.10, respectively). In contrast, symmetry behaviour (*h*^2^ = 0.39 ± 0.03) was significantly correlated with TS PRS (*p* = 0.02) and not with OCD and ADHD (*p* = 0.18 and *p* = 0.26, respectively).

There have also been noteworthy advances in the understanding of the neuronal circuitry of TS. The role of the cortico-striatal-thalamo-cortical circuits (CSTC) in TS has been emphasized^17^, although data on changes in the volume and function of specific brain regions in the CSTC in individuals with TS is less consistent. Lower bilateral nucleus caudate volumes^18,19^, inferior occipital volumes^20^, prefrontal cortex volumes^20,21^, corpus callosum volumes and decreased white matter connectivity^22,23^ have been observed in children with TS. Greater grey matter volumes have also been observed in the thalamus^21,24^, hypothalamus and midbrain among children and adults with TS^21,25^. Amygdalar volume has been reported to be greater in children and lower in adulthood^25^.

Little work to date has, however, focused on pleiotropy or concordance of genetic risk for TS, and genetic variants that influence subcortical brain volume. Pleiotropy refers to a SNP that affects both phenotypes, regardless of whether the effect direction is the same for both. Concordance, however, requires that the SNP has the same direction of effect for both phenotypes. The Enhancing Neuroimaging Genetics through Meta-analysis (ENIGMA) consortium recently performed a GWAS of structural brain MRI scans of 30,717 individuals^26^. The ENIGMA subcortical brain volumes study identified novel genetic variants associated with the volumes of the putamen and caudate nucleus^26^ and subsequently detected an overlap with OCD risk variants^27^. ENIGMA provides an opportunity to examine the relationship between GWAS data in TS with the genetic contributions to regional brain volumes. Here we aim to assess genetic concordance for TS and specific symptom profiles (i.e., social disinhibition and symmetry behaviour) with the volume of relevant subcortical and intracranial brain regions. We used summary statistics from the ENIGMA subcortical and intracranial brain volumes GWAS^26^ and the most recent PGC-TS GWAS^5^.

## Methods

### Description of original association studies

We obtained summary statistics of adult European ancestry participants (4644 cases and 8695 controls) and 9,076,550 SNPs from the most recent PGC-TS GWAS^5^, including unpublished data. Approximately half of this cohort also had either comorbid OCD or ADHD. A subset of cases had information regarding symmetry behaviour (*n* = 1419) and social disinhibition (*n* = 1414) symptom classes. Participants were diagnosed using the DSM-IV-TR^28^ by trained clinicians. In addition, we used GWAS summary statistics from the ENIGMA Consortium meta-analysis of subcortical brain volumes across 50 cohorts, including MRI scans of 30,717 individuals and 9,702,043 SNPs^26^. This cohort consisted of healthy controls (79%) as well as patients (21%) diagnosed with neuropsychiatric disorders (including anxiety disorders, Alzheimer’s disease, ADHD, major depression, bipolar disorder, epilepsy and schizophrenia). A direct comparison of the GWAS summary statistics between the full ENIGMA results (including patients) and a subset of ENIGMA results (excluding patients) showed that they were very highly correlated (Pearson’s *r* > 0.99) for all brain traits^26^. Prior to the analyses here, we verified that there was no cohort overlap between the TS and brain volume GWASs and therefore individual overlap was likely to be minimal, if any at all. The brain volume GWAS data comprised GWASs of seven subcortical brain volumes (nucleus accumbens, amygdala, caudate nucleus, hippocampus, globus pallidus, putamen, thalamus) and total intracranial volume (ICV). GWAS test statistics were genome-controlled to adjust for spurious inflation factors. All cohort studies were approved by a local ethics board prior to subject recruitment and all subjects gave written, informed consent before participating.

### Post-processing of genetic data

To statistically compare the TS and brain volume GWASs, we used the 7,682,991 SNPs that passed quality control and filtering rules in all datasets. With these data, we performed a clumping procedure in PLINK^29^ to identify an independent SNP from every linkage disequilibrium (LD) block across the genome. The clumping procedure was performed separately for each of the eight brain volume GWASs using a 500 kb window, with SNPs in LD (*r*^2^ > 0.2), in the European reference samples from the 1000 Genome Project (Phase 1, version 3). The SNP with the lowest *p*-value within each LD block was selected as the index SNP representing that LD block and all other SNPs in the LD block were dropped from the analysis. The result, after applying the clumping procedure, was a total of eight independent sets of SNPs representing the total variation explained across the genome, conditioned on the significance in each brain volume GWAS. For each of these eight sets of SNPs, we then determined the corresponding TS GWAS test statistic for each independent index SNP and used these datasets for the subsequent analyses.

### Tests of pleiotropy and concordance

We used SNP effect concordance analysis (SECA)^30^ (see Nyholt 2014 for details of the SECA analysis) to test for genetic pleiotropy—the same SNP affecting two traits—and concordance—the agreement in SNP effect directions across these two traits—between TS, social disinhibition or symmetry behaviour and all seven subcortical structures and ICV.

### Conditional false discovery rate to detect TS, social disinhibition or symmetry behaviour risk variants

We also examined if conditioning the results of the TS GWAS on genetic variants that influence subcortical brain volume (TS | subcortical brain volume) could improve our ability to detect variants associated with TS^31,32^. At this sample size, the analyses were underpowered to investigate the symptom dimensions separately. For a given brain volume phenotype, we selected subsets of SNPs at 14 false discovery rate (FDR) thresholds (*q*-values ≤ 1 × 10^−5^, 1 × 10^−4^, 1 × 10^−3^, 0.01, 0.1, 0.2, 0.3, 0.4, 0.5, 0.6, 0.7, 0.8, 0.9, 1) and looked up the corresponding *p*-values for each SNP subset in the TS GWAS and the social disinhibition and symmetry behaviour symptom clusters. Next, we applied the FDR method^33^ to each subset of *p*-values in the TS GWAS and the social disinhibition and symmetry behaviour symptom clusters. Individual SNPs were considered significant if the *p*-value was lower than the significance threshold, allowing for an FDR of 5%, conditioned on any subset of SNPs from the brain volume GWASs. The LD-pruned data were required for the conditional FDR SNP analysis because regions with varying amounts of SNPs within an LD block can affect the ranking and re-ranking of SNPs under the conditional models. However, the chosen SNP included in the model is likely just a “proxy” for SNPs in the LD block and should not necessarily be considered a causal variant or even the most significant SNP in terms of its overlap between traits.

Stratified true discovery rate (TDR) plots were constructed by subsetting SNPs based on associations with a secondary trait (i.e. subcortical brain volumes), and generating TDR plots separately for each subset of SNPs based on their association with the main trait of interest (i.e. TS). The SNPs that had reached at least marginal significance thresholds for the relevant subcortical brain regions were selected for inclusion in the plots. The selected *p*-value thresholds were 1.0, 0.1, 0.01 and 0.001, which were log-transformed. A conservative measure of TDR is calculated for each SNP as 1 – (*p*/*q*), where *p* represents the *p*-value of a SNP’s association with the primary trait of interest (TS), and *q* represents the empirical conditional cumulative distribution function *q* (TS | subcortical brain region)^34^.

### Estimating genetic correlation using LD score regression

In order to replicate significant findings from our primary analysis with SECA, we used an alternative method, LD score regression (LDSR), which estimates a genetic correlation between two trait pairs based on the GWAS summary statistics of each trait analysed separately^35,36^. LDSR estimates a genetic correlation with a fitted linear model of Z-scores obtained from the product of significance statistics for each SNP in a given set of GWAS results compared to the level of linkage disequilibrium at a given SNP. SNPs in high LD are expected to have high Z-scores in polygenic traits with common genetic overlap^35^. We used the ldsc program (https://github.com/bulik/ldsc) to perform LDSR following the methods outlined in Bulik-Sullivan et al., 2015^36^. LDSR was underpowered at this sample size to analyse the amygdala, as well as the TS symptom clusters. Therefore, LDSR was only used to test for an association with the broader TS phenotype and the remaining seven brain regions of interest. Given the number of tests performed, we set a Bonferroni corrected significance level at p* = 0.05/48 = 1.042 × 10^−3^ to account for the three traits tested (TS, social disinhibition and symmetry behaviour), the eight brain volumes and the two tests performed (SECA and LDSR).

## Results

### Evidence for pleiotropy between brain volume and TS and related symptom cluster risk variants

We found significant evidence of global pleiotropy—same SNP, regardless of effect direction—between variants that infer risk for the broader TS phenotype and variants that are associated with lower putamen (Table 1, *p* *=* 2 × 10^−4^) and caudate volumes (Table 1, *p* *=* 4 × 10^−4^). Further, we found nominally significant (*p* < 0.05) evidence of pleiotropy between TS risk variants and variants associated with lower ICV, accumbens, pallidum, and thalamus volumes and greater hippocampus volume (Table 1). No evidence for global pleiotropy was found for either the social disinhibition (Table 2) or symmetry behaviour (Table 3) symptom clusters.

**Table 1:** SECA results for TS whole cohort

Trait 1	Trait 2	*p*-value pleiotropy	CI pleiotropy	*p*-value concordance	CI concordance	Direction
TS	Intracranial volume	0.001**	0.001–0.002	0.022**	0.019–0.025	–
	Accumbens	0.007**	0.006–0.009	0.054*	0.049–0.058	–
	Amygdala	1	1–1	0.048**	0.044–0.053	+
	Caudate	4 × 10^−4^***	1 × 10^−4^–0.001	0.016**	0.013–0.018	–
	Hippocampus	0.009**	0.007–0.011	0.07*	0.065–0.075	+
	Pallidum	0.006**	0.004–0.007	0.257	0.249–0.266	–
	Putamen	2 × 10^−4^***	5.48 × 10^−5^–0.007	1	1–1	–
	Thalamus	0.009**	0.008–0.012	3 × 10^−4^***	1 × 10^−4^–0.001	–

*TS* Tourette’s syndrome, *CI* confidence interval, Bonferroni corrected *p-*value = 0.05/48 = 1.042 × 10^−3^

*Trending significance (*p* < 0.1)

**Nominally significant (*p* < 0.05)

***Significant

**Table 2:** SECA results for TS social disinhibition endophenotype

Trait 1	Trait 2	*p*-value pleiotropy	CI pleiotropy	*p*-value concordance	CI concordance	Direction
TS	Intracranial volume	1	1–1	1	1–1	+
	Accumbens	0.31	0.301–0.319	0.002**	0.002–0.003	–
	Amygdala	0.166	0.159–0.173	0.084*	0.079–0.09	–
	Caudate	1	1–1	0.166	0.159–0.173	–
	Hippocampus	1	1–1	0.099*	0.093–0.105	–
	Pallidum	1	1–1	0.047**	0.043–0.051	+
	Putamen	1	1–1	0.194	0.186–0.201	–
	Thalamus	1	1–1	1 × 10^−3^***	5.13e−06–0.001	–

*TS* Tourette’s syndrome, *CI* confidence interval, Bonferroni corrected *p-*value = 0.05/48 = 1.042 × 10^−3^

*Trending significance (*p* < 0.1)

**Nominally significant (*p* < 0.05)

***Significant

**Table 3:** SECA results for TS symmetry endophenotype

Trait 1	Trait 2	*p*-value pleiotropy	CI pleiotropy	*p*-value concordance	CI concordance	Direction
TS	Intracranial volume	1	1–1	0.191	0.183–0.198	–
	Accumbens	0.304	0.296–0.314	0.263	0.255–0.272	–
	Amygdala	0.298	0.289–0.307	0.002**	0.001–0.003	–
	Caudate	0.304	0.295–0.313	0.017**	0.015–0.02	+
	Hippocampus	1	1–1	0.07*	0.065–0.075	+
	Pallidum	1	1–1	0.417	0.408–0.427	+
	Putamen	0.313	0.304–0.322	0.001***	0–0.001	+
	Thalamus	1	1–1	1	1–1	–

*TS* Tourette’s syndrome, *CI* confidence interval, Bonferroni corrected *p-*value = 0.05/48 = 1.042 × 10^−3^

*Trending significance (*p* < 0.1)

**Nominally significant (*p* < 0.05)

***Significant

### Evidence for concordance between brain volumes and TS and related symptom clusters risk variants

We found significant evidence of negative concordance—same SNP, same direction of effect—between TS and thalamus volume (Table 1, *p* *=* 3 × 10^−4^), indicating an association between TS genetic risk and lower thalamus volume. Further, we found nominally significant (*p* < 0.05) negative concordance between ICV and caudate volumes with TS (Table 1). Nominally significant positive concordance was found between the amygdala and TS. Significant negative concordance was also identified between the social disinhibition behaviour symptom cluster and the thalamus (Table 2, *p* *=* 1 × 10^−3^) as well as marginally significant negative concordance with the accumbens (*p* *=* 2.3 × 10^−3^) and positive concordance with the pallidum (*p* *=* 0.47 × 10^−2^). Significant positive concordance was also identified between putamen volume and symmetry behaviour (Table 3, *p* *=* 7 × 10^−4^). Evidence for marginally significant negative concordance between this symptom cluster and the amygdala (*p* *=* 2 × 10^−3^) and positive concordance with the caudate (*p* *=* 1.7 × 10^−2^) were also observed.

### Replication of subcortical brain volumes and TS genetic risk overlap using LDSR

Replication using LDSR lent trending support for an association between TS risk and the thalamus (Table 4, *p* *=* 5.85 × 10^−2^), although this was not significant. No association between TS and pallidum volume was observed.

**Table 4:** LDSR results for TS whole cohort and brain volume overlap

Trait 1	Trait 2	rg	SE	95% CI	*p*-value
TS	Intracranial volume	−0.122	0.075	−0.269–0.025	0.106
	Accumbens	−0.428	0.582	−1.568–0.712	0.462
	Amygdala	–	–	–	–
	Caudate	−0.037	0.087	−0.208–0.134	0.672
	Hippocampus	−0.015	0.097	−0.205–0.175	0.878
	Pallidum	−0.002	0.106	−0.21–0.206	0.983
	Putamen	−0.023	0.091	−0.201–0.155	0.801
	Thalamus	−0.227	0.12	−0.462–0.008	0.059*

*TS* Tourette’s syndrome, *CI* confidence interval, Bonferroni corrected *p-*value = 0.05/48 = 1.042 × 10^−3^

*Trending significance (*p* < 0.1)

### Genetic variants influencing subcortical brain volumes provide improved ability to detect TS risk variants

We performed a conditional FDR analysis, conditioning the TS risk variants on each of the eight brain volume GWASs (Table 5). When conditioning the TS analysis on variants that influence ICV, rs2708146 (*q* = 0.046) and rs72853320 (*q* *=* 0.035) were significantly associated with both traits. Conditioning TS on the hippocampus also revealed an association between rs1922786 (*q* = 0.001). Each of these variants account for <1% of the variance observed in TS, as well as in each of the associated brain region volumes. No significant associations (*q* < 0.05) were identified when conditioning TS on the other six brain GWAS.

**Table 5:** Conditional FDR results for TS and endophenotypes with brain volume overlap

Structure	SNP	Chr	BP	EA	NEA	Freq	Nearest gene	Distance to gene (bp)	Effect in brain ± standard error	*p*-value in brain	% Variance explained in brain	Effect in TS ± standard error	*p*-value in TS	% Variance explained in TS	*q*-value
Intracranial volume	rs2708146	2	58955953	A	G	0.5428	LINC01122	Intronic	484.913 ± 1901.256	0.799	5.72 × 10^−4^	0.141 ± 0.027	1.3 × 10^−7^	0.2089	0.046
	rs72853320	6	36623338	A	G	0.1331	RNU1-88P	15889	−3769.398 ± 2836.113	0.184	0.0155	0.205 ± 0.04	2.42 × 10^−7^	0.1997	0.035
Hippocampus	rs1922786	2	58863573	A	G	0.6556	LINC01122	Intronic	−17.527 ± 4.895	0	9.62 × 10^−5^	0.14 ± 0.028	7.93 × 10^−7^	0.1828	0.001

The chromosome (Chr) and base pair (BP) are given in h19b37 coordinates. The effect in brain and effect in TS (Tourette’s syndrome) are both given in terms of the effect allele (EA). The non-effect allele (NEA) is also shown. The allele frequency (Freq) corresponds to the effect allele. Tagging SNP corresponds to the most significant variant in a given LD block (if different from the SNP chosen based on clumping in the brain volume GWAS). LINC01122 = long intergenic non-protein coding RNA 1122, RNU1-88P = RNA, U1 small nuclear 88, pseudogene

**Fig. 1:**
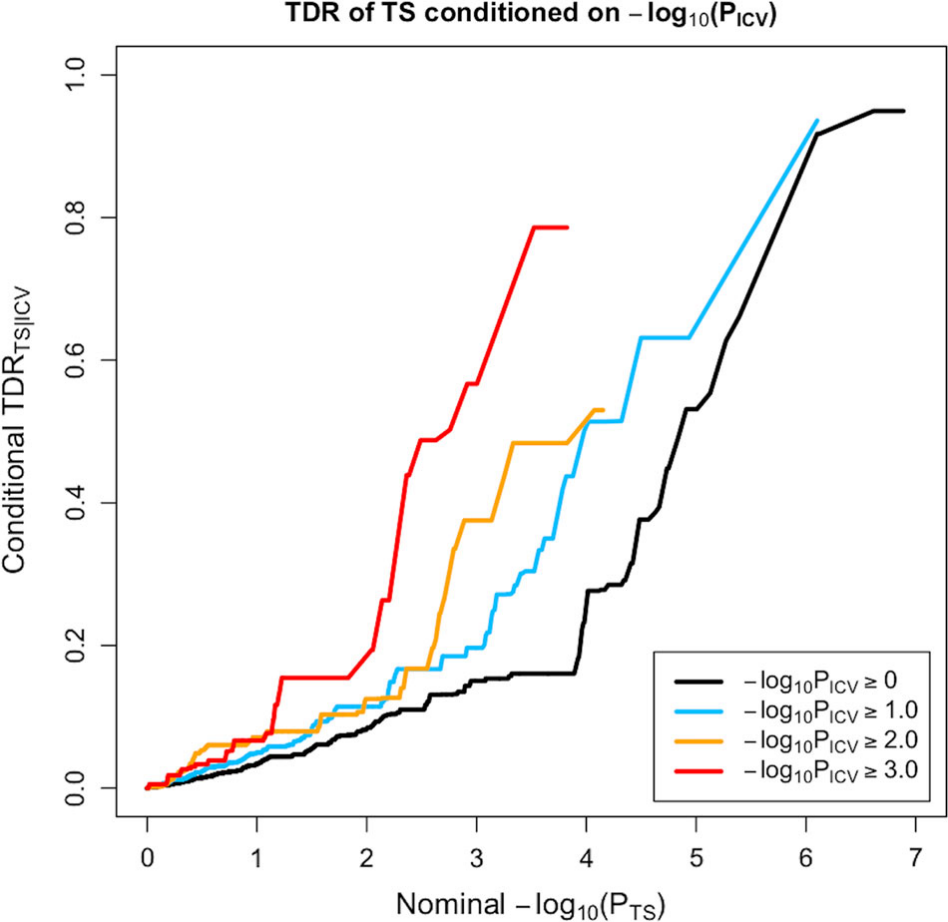
QQ plot of TS conditioned on ICV. The stratified true discovery rate (TDR) plot illustrates the increase in TDR associated with increased pleiotropic enrichment in TS conditional on nominal ICV *p*-values

**Fig. 2:**
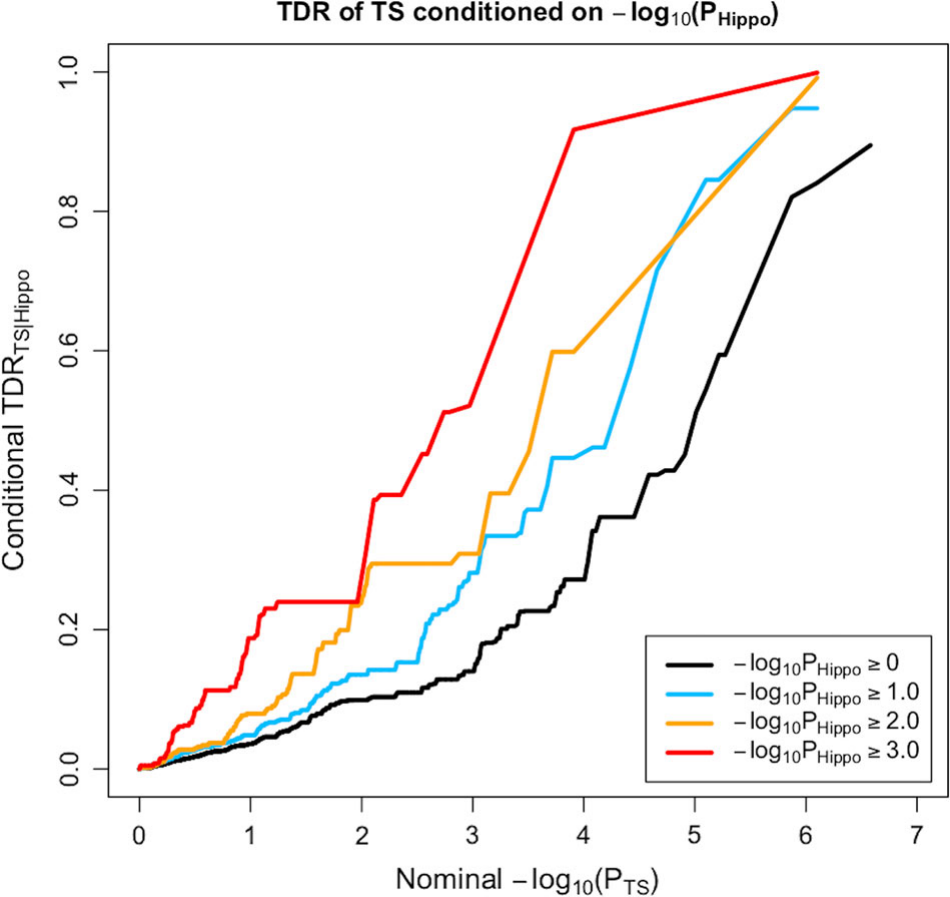
QQ plot of TS conditioned on hippocampus volume. The stratified true discovery rate (TDR) plot illustrates the increase in TDR associated with increased pleiotropic enrichment in TS conditional on nominal hippocampus volume *p*-values

The conditional TDR reflects the posterior probability that a given SNP is truly associated with the first phenotype (i.e. TS) given that the *p*-values for both phenotypes are as small or smaller than the observed *p*-values. The TDR plots show an increase in TDR associated with increased pleiotropic enrichment in TS conditional on nominal *p*-values for (i) ICV (Fig. 1) and (ii) hippocampus volume (Fig. 2). The successive leftward shifts for decreasing nominal *p*-value thresholds of both ICV and hippocampus volumes indicate that the proportion of SNPs with non-null effects on TS varies considerably across various levels of association with each of these subcortical brain volumes.

## Discussion

Of the eight brain traits investigated using SECA, associations were found between genetic risk for TS and for lower thalamus, putamen and caudate volumes. These analyses of the clinical TS phenotype were further supported by SECA findings using symptom-based phenotypes; there was a significant association between genetic risk for lower thalamus volume and for the social disinhibition symptom cluster as well as a significant association between genetic risk for greater putamen volume and for the symmetry symptom cluster. Further, three SNPs were associated with both TS and the volumes of the hippocampus and ICV.

Associations of genetic risk for TS with genetic risk for alterations in thalamus and striatum volumes is consistent with the emphasis of neuroanatomical models of TS on these structures^37,38^. The thalamus has widespread projections affecting many aspects of cognition and motor function^39^, and is a target for deep brain stimulation in the treatment of refractory TS^40^. While greater thalamus volumes have been reported in TS^21,24^, lower thalamus volumes have been identified in paediatric patients with TS^41^. The dorsal striatum is also involved in motor function^42^, as well as various types of learning^43,44^, inhibitory control of action, and reward systems^45^. Evidence for both greater^46,47^ and lower putamen volumes^18,21,48^ has been reported in both children and adults with TS, with lower putamen volumes reported in TS with comorbid OCD^18^. Lower caudate volumes in adults and children have been associated with TS^18,48^, particularly apparent in individuals with comorbid ADHD^18^, and are associated with tic severity and OCD symptoms^48^.

We also report a significant association between genetic risk for lower thalamus volume and genetic risk for social disinhibition symptoms. Social disinhibition has been hypothesized to be an endophenotype that is relevant to OCD, TS and ADHD, reflecting deficits in top-down cognitive control across all of these conditions^16^. Given the involvement of the thalamus in many aspects of cognition, our findings are consistent with this hypothesis^39^.

The association of genetic risk for greater putamen volume and genetic risk for symmetry symptoms suggests that the putamen may play a particularly important role in symmetry behaviours. CTSC circuitry certainly plays an important role in TS^38^ and it has been suggested that more complex tics—such as those involving symmetry behaviours—may be mediated by ventral striatal circuits^49^ that are also involved in OCD^27,50^. However, not all data are consistent; symmetry symptoms in TS may not share a significant degree of genetic overlap with OCD^16^ and findings from neuroimaging of symmetry symptoms in OCD have not emphasized striatal regions^51,52^.

The analyses here complement prior work on OCD, where we found significant concordance between OCD risk variants and variants that are related to greater putamen volume (*p* = 8.0 × 10^−4^)^27^. In addition, the CSTC has been implicated in both disorders^38,53^. Our previous study on OCD did, however, also identify significant concordance between greater nucleus accumbens volume and OCD and conditional FDR only revealed significant associations with OCD when conditioned on putamen, amygdala and thalamus volumes^27^. Differences between OCD and TS are consistent with genetic analyses which have emphasized that despite their relatedness, these conditions have different genetic architectures^4,54^. The findings here arguably support the proposal in the eleventh revision of the International Classification of Diseases (ICD-11) to classify TS as both a neurodevelopmental disorder and as an obsessive-compulsive related disorder (OCRD)^1,55^.

The analysis here also complements previous work on ADHD; for example, the largest subcortical brain volume study of ADHD to date found lower putamen volumes as well as other alterations in this disorder^56^. Notably, although the putamen is also implicated in the TS symmetry cluster, the effect direction is opposite to that observed in ADHD^56^. Additional work on neuroimaging of children with TS may help clarify these differences.

The three variants associated with TS after conditioning on hippocampal (rs1922786) and ICV (rs2708146 and rs72853320) volumes have not previously been associated with a neuropsychiatric phenotype. Both SNPs, rs2708146 and rs1922786, are intronic variants located within the long intergenic non-protein coding RNA 1122 (*LINC01122*) gene on chromosome 2. The SNP, rs72853320, is an intergenic variant closest to the RNA U1 small nuclear 88 pseudogene (*RNU1*-88P) on chromosome 6. To date, little is known about the function of these genes, which are expressed in a broad range of tissue types, including the brain (https://www.ensembl.org/index.html, accessed on 5 April 2018). Other variants within *LINC01122* have previously been associated with red blood cell count, mean corpuscular haemoglobin and mean corpuscular volume in a GWAS investigating blood cell phenotypes in the UK Biobank^57^. The study of blood cell phenotypes identified a total of 2706 variants associated with these phenotypes, located within or near genes that are involved in pathways that have been implicated in schizophrenia, autoimmune diseases and cardiovascular disorders^57^. Further, each of the three variants identified in the current study only accounted for <1% of the variance observed in TS, as well as in each of the brain region volumes. Better powered studies are needed to address this issue.

Several limitations of this work should be emphasized. First, the LDSR analysis only lends trending, but not significant, support for the association between the thalamus and TS. LDSR was further underpowered to perform all the tests of brain volume with the TS symptom clusters. LDSR is ideally suited for datasets with more than 3000 samples, which was not the case for the TS symptom dimensions^58^. Estimating partitioned heritability typically requires datasets with at least 5000 samples^58^. Approaches such as genome-wide complex trait analysis may be more applicable in future studies with these sample sizes, when individual-level data are available^58^. LDSR requires larger datasets compared to SECA and cFDR as it determines genetic correlation in one direction of effect, whereas the latter techniques use the absolute size of effect and are able to distinguish whether the effect allele of a SNP confers risk for both traits or whether the effect directions are opposite for the traits^30,32,36^. This also contributes to differences in the findings obtained across these techniques. Validation in an even larger cohort is necessary. Second, the TS GWAS that was used in this study had low power^5^ and may be susceptible to random variation. This is currently the largest dataset available for TS and as this dataset grows, replication studies will be possible. Third, the analysis could be biased if overlapping participants were present in the studies contributing to the consortia. We verified that the cohorts as whole did not overlap and individual overlap is, therefore, likely to be minimal. Fourth, the ENIGMA GWASs of brain volumes contain cohorts with healthy controls as well as patients diagnosed with neuropsychiatric disorders (including anxiety, Alzheimer’s disease, ADHD, major depression, bipolar disorder, epilepsy and schizophrenia), which may bias the interpretation of our findings and how they relate to TS. However, a direct comparison of the GWAS summary statistics between the full ENIGMA results (including patients) and a subset of ENIGMA results (excluding patients) showed that they were very highly correlated (*r*^2^ > 0.99) for all brain traits^26^. This suggests that the pattern of effects in the brain volume GWAS is not likely driven by disease status. Fifth, approximately half of the TS cohort also has comorbid OCD. It is possible that stronger and more specific associations may be revealed in a cohort with TS only. Sixth, this study only investigated samples of European ancestry and the results are possibly only applicable to this population. Replication studies using other population groups are needed^59^. Seventh, the relationship between gene variants influencing brain volume and neuropsychiatric risk may be influenced by a range of confounders, including environmental factors such as stressors and medication effects, which may have effects on brain volume and disease risk independent of genetics. Discovering the pathway by which gene variants influencing brain volume also create risk for TS and its symptom clusters is therefore susceptible to influence by environmental factors, which might obscure genetic relationships. Future work on gene by environment interactions would be useful to clarify this. However, an endeavour to find the genetic overlap between brain volume and disorder risk using the largest datasets to date is still worthwhile, as it can potentially provide insights into the disorder.

In conclusion, this study implicated genetic overlap between genetic variants influencing the volumes of the thalamus, putamen and caudate in TS and its symptom dimensions. Further, this study identified three SNPs associated with TS and volumes of the hippocampus and ICV. Indeed, these data are the first to show an overlap between risk for genes for TS and for brain circuitry. The correlations with putamen and thalamus volumes are consistent with a broad range of previous neuroimaging work. Emerging collaborations and consortia, such as ENIGMA-TS, aim to continue to increase sample size, which will enhance statistical power in future iterations of this analysis. Additional work focusing on a range of other methodologies to assess genetic overlap may also be useful, following along the lines of recent work in schizophrenia^60–62^. Such studies have used partitioning-based heritability analysis^63^ and conjunction analysis^64^ to identify genetic variants associated with both schizophrenia risk and altered brain volumes. These approaches may also be useful in future work on TS and its symptom dimensions.
